# Development and upgrading of public primary healthcare facilities with essential surgical services infrastructure: a strategy towards achieving universal health coverage in Tanzania

**DOI:** 10.1186/s12913-020-5057-2

**Published:** 2020-03-17

**Authors:** Ntuli A. Kapologwe, John G. Meara, James T. Kengia, Yusuph Sonda, Dorothy Gwajima, Shehnaz Alidina, Albino Kalolo

**Affiliations:** 1 President’s Office – Regional Administration and Local Government (Directorate of Health, Social Welfare and Nutrition Services), P.O Box 1923, Dodoma, Tanzania; 2 Program in Global Surgery and Social Change, Harvard Medical School, Boston, MA USA; 3 Department of Plastic and Oral Surgery, Boston Children’s Hospital, Boston, MA USA; 4 Department of Public Health, St. Francis University College of Health and Allied Sciences, P.O Box 175, Ifakara, Tanzania

**Keywords:** Infrastructure, Primary health facilities, Safe surgery, Universal health coverage

## Abstract

**Background:**

Infrastructure development and upgrading to support safe surgical services in primary health care facilities is an important step in the journey towards achieving Universal Health Coverage (UHC). Quality health service provision together with equitable geographic access and service delivery are important components that constitute UHC. Tanzania has been investing in infrastructure development to offer essential safe surgery close to communities at affordable costs while ensuring better outcomes. This study aimed to understand the public sector’s efforts to improve the infrastructure of primary health facilities between 2005 and 2019. We assessed the construction rates, geographic coverage, and physical status of each facility, surgical safety and services rendered in public primary health facilities.

**Methods:**

Data was collected from existing policy reports, the Services Availability and Readiness Assessment (SARA) tool (physical status), the Health Facility Registry (HFR), implementation reports on infrastructure development from the 26 regions and 185 district councils across the country (covering assessment of physical infrastructure, waste management systems and inventories for ambulances) and Comprehensive Emergence Obstetric Care (CEMONC) signal functions assessment tool. Data was descriptively analyzed so as to understand the distribution of primary health care facilities and their status (old, new, upgraded, under construction, renovated and equipped), and the service provided, including essential surgical services.

**Results:**

Of 5072 (518 are Health Centers and 4554 are Dispensaries) existing public primary health care facilities, the majority (46%) had a physical status of A (good state), 33% (1693) had physical status of B (minor renovation needed) and the remaining facilities had physical status of C up to F (needing major renovation). About 33% (1673) of all health facilities had piped water and 5.1% had landline telecommunication system. Between 2015 and August 2019, a total of 419 (8.3%) health facilities (Consisting of 350 health centers and 69 District Council Hospitals) were either renovated or constructed and equipped to offer safe surgery services. Of all Health Centers only 115 (22.2%) were offering the CEMONC services. Of these 115 health facilities, only 20 (17.4%) were offering the CEMONC services with all 9 - signal functions and only 17.4% had facilities that are offering safe blood transfusion services.

**Conclusion:**

This study indicates that between 2015 and 2019 there has been improvement in physical status of primary health facilities as a result constructions, upgrading and equipping the facilities to offer safe surgery and related diagnostic services. Despite the achievements, still there is a high demand for good physical statuses and functioning of primary health facilities with capacity to offer essential and safe surgical services in the country also as an important strategy towards achieving UHC. This is also inline with the National Surgical, Obstetrics and Anesthesia plan (NSOAP).

## Background

Achieving Universal Health coverage (UHC) is a top priority of the health reform agenda in many countries [1]. The World Health Organization (WHO) defines Universal Health Coverage (UHC) as the desired performance of a successful health system, whereby all people are provided with access to needed health services (including prevention, promotion, treatment, rehabilitation and palliation) of sufficient quality to be effective without causing financial hardship [1].

UHC consists of three inter-related components: (1) the full spectrum of high-quality, needs-based essential healthcare services, (2) protection from financial hardship due to out-of-pocket payments for health services, and (3) coverage for the entire population [2]. These are measured based on (1) health service coverage, (2) financial protection coverage, and (3) equity in coverage [2]. Achieving UHC entails implementing interventions that target both supply- and demand-side of the health care system. In the supply side efforts that focus on improving quality of services rendered to the people, infrastructural development and having adequate skilled personnel manned at the primary health facility levels could hasten attainment of UHC whereas on the demand side efforts that capitalize on providing feedback to the health service providers through established social accountability framework like the community score cards and ensuring that health services are people centered are necessary for UHC attainment. Advancing progress towards universal health coverage should also be accompanied by research [3]. However, most of the research conducted is on how financial mechanisms can contribute towards achieving the UHC and very little research has been conducted to see how infrastructure development (particularly in the primary health settings of low- and middle-income countries) contributes to achieving UHC.

Infrastructure development is an important component of a well-functioning healthcare system. Health system infrastructure ranges from the physical facilities, information systems to medical equipment and also involves construction of new infrastructure as a strategy to achieving UHC [4]. To provide quality health services required for universal health coverage, health facilities should be structured to meet health care needs and equipped with utilities such as electricity, water and skilled health workforce and also to construct or renovate primary health facilities that are able to offer quality services [5, 6]. Furthermore, improvement in geographical access of health services provision through strategic infrastructure development strengthens the referral system, increase service utilization and, in so doing, improve health outcome indicators at individual and community level. Existing evidence also attest that availability of high-quality basic amenities leads to an increase in utilization of the health care system [7, 8].

In Tanzania, given the rapid population growth (around 3.1% with the total fertility rate of 5.0) projected to reach 89.2 million people by 2035 [9] which is approximately two times the current population, infrastructure development particularly of the primary health facilities should match with the fast growing population to limit the increase in mortality rates (in particular neonatal and maternal mortality rates). Quality Maternal health services is one of the key priorities of the health sector in Tanzania, and was recognized as permanent agenda in all of its strategies including the Health Sector Strategic Plan III and IV [10], National Surgery, Obstetric and Anesthesia Plan - NSOAP) [11], and Development Vision 2025 will be realized only if health facility infrastructure is improved and equipped with all required inputs.

Primary health care (PHC) is an important entry point into the healthcare system by majority (95%) of people [12] in Tanzania; it is therefore important to invest in quality infrastructure development to improve the health service utilization and quality of services rendered to the population. Primary Health Care (PHC) in Tanzania was first conceived in 1967 during the Arusha Declaration. The Arusha Declaration laid down ideas similar to those of PHC through the “ujamaa” policy that advocated for provision of free health care to all people and the Decentralization Act of 1972 [13] advanced the idea resulting in the establishment of numerous health facilities throughout the country. Although the decentralization policy of 1972, failed, LGAs were reintroduced in 1981 [14]. The Alma Ata Declaration on PHC in 1978 and the Astana Declarations of 2018 [15, 16] both emphasized on the role of primary health care in service provision to achieve an ambitious goal of health for all hence universal health coverage. Since independence (1961), Tanzanian health policies have established a clear objective of achieving primary health care for all, by designing and implementing initiatives for increasing access to health care; including efforts to ensure that a majority of people lives within 5 Kms of health facilities [12]. Thereafter, a number of reforms have been implemented in the primary health care and include (to mention few); the Health Sector Reform of 1994 that was focusing on addressed improving access, quality and efficiency in health service delivery after structural adjustment program of 1993 [17]. The Medical stores department (MSD) in 1993 and Prime Vendor System (PVS) in 2018 to strengthen supply chain for essential medicines in primary health facilities through a public private partnership (PPP) arrangement, Health financing reforms through introduction of the Community Health Fund in 1996 and its improved version (iCHF) in 2011 and introduction of Direct Health Facility Financing (DHFF) in 2017 [18, 19].

The targeted infrastructure development program for primary health facilities began in 2007, through a 10-year primary healthcare development programme (2007–2017) [20]. The Primary Health Care Services Development Programme (PHSDP), also locally known as “*Mpango wa Maendeleo wa Afya ya Msingi (MMAM)”* has been instrumental in the development of infrastructure and other health system inputs such as human resources, medical equipment and supplies, but also strengthening of the referral systems across the country. However, to date, the performance of this program remains largely unknown, as there is no publicly available evaluation report to offer information on the implementation and outcomes of the program.

Star rating approach for primary health facilities is another strategy to improve primary health care infrastructure that rates the primary health facilities in relation to set scores and takes appropriate actions per score obtained by the facility. Introduced in 2015 by Ministry of Health, Community Development, Gender, Elderly and Children, star rating is based on the minimum score out of four domains (A = Facility management and staff performance (20); B = Service charters & accountability (30); C = Safe and conducive facilities (20); D = Quality of care & services (30). Zero-star facilities are those scoring less than 20% in any one of the four domains [21]. There are percentages scores attached to each grade so as to classify facilities into 5 grades starting with 0 star rated facility. For 0 star the point scored are between 0 and below 20; For 1 star is between 20 and less than 40%; For 2 stars is between 40 and less than 60; For 3 starts is between 60 and less than 80; For 4 stars is between 80 and less than 90 and for five stars is between 90 and 100%. Since 2015, Star rating has been used to grade all the primary health facilities in Tanzania, for example in 2015 less than 2% of primary healthcare facilities (131/6993) met the required quality standard of three-stars or above. A three-star rating is considered a fair point at which to make further investments for certification according to recognized international schemes, such as international accreditation program (IAP) [22]. About 1 in 8 facilities (13%) achieved two-star rating. Just over half (51%) of all assessed facilities were rated one-star, and about one-third were rated zero-star (34%), with the latter category requiring urgent attention [20]. The repeated star rating assessment exercise of 2018 showed some improvement in the areas of human resources for health and health commodities although still there is uneven distribution of health care facilities and poor qualities of amenities, the findings showed that 456 (6%) of primary health facilities scored 0; 2396 (33%) scored 1 star; 3067 (42%) score 2 stars; 1276 (18%) scored 3 stars; 94 (1%) scored 4 stars and none of the facilities scored 5 stars [21].

Some similar assessment of the infrastructure in public health facilities found that; the physical condition of the health facility buildings (infrastructure) was poor [21, 23]. The assessments included the Service Availability and Readiness Assessment (SARA) of 2012 and Tanzania Service Provision Assessment (TSPA) of 2014/2015. More than 50% of facilities required urgent major renovation or complete reconstruction. There was inadequate space for service provision as more than 60% of the health facilities did not have the required number of rooms based on the standards defined by the Ministry of Health, Community Development, Gender, Elderly and Children (MoHCDGEC) [24].

In the Joint Annual Health Sector Review meeting in December 2016, between PORALG and MoHCDGEC in collaboration with the different stakeholders including Development Partners it was agreed that; there should be a deliberate renovation and construction of some primary health facilities as a strategy to improve the physical status of dilapidated health facility buildings [25]. Thereafter, a team with multiple stakeholders led by the President’s Office, conducted supportive supervision and cost analysis to ascertain the magnitude of construction/ rehabilitation of primary health care facilities to guide the improvement plan. After the visits, the team documented deteriorating physical infrastructure, uneven distribution of health facilities, and also challenges in the referral system. This report was in concordance with other reports such as that presented during the annual Regional Medical Officer’s (RMOs) and District Medical Officer’s (DMOs) meeting in the same year and other survey and supervision findings such as un-availability of facilities for safe surgeries, no or inadequate laboratory for safe blood and poor waste management [25]. The report from the team visit lead to a resolution to renovate all health facilities based on the following criteria: 1) a distance from the district head office of more than 20 km, 2) Health facilities with a physical state of C and D (Table 1), 3) Inadequate availability of utilities, such as water and electricity, 4) The status of transportation methods to enhance the referral system 5) Areas with a catchment population of more than 10,000 people and 6) Health centers with no operating theatre. The aim this time was to equip these facilities so that they can also offer safe surgeries to people in need.

**Table 1:** Health facility physical status assessment tool

S/N	Physical status	Definition	Checklist
1.	A	Good	A building with nothing to be fixed
2.	B	Minor renovation	1 Visible narrow cracks on concrete surfaces (crack width < 0.2 mm) 2 Damage to the door locks 3 Damage to the window glass
3.	C	Major renovation	1. Visible wide cracks on concrete surfaces (crack width about 0.2 mm – 1.0 mm) 2. Foundation damage 3. Significant damage to concrete, with exposed reinforcing bars 4. Spalling of concrete surfaces (crack width of more than 2.0 mm)
4.	D	Demolition and re-construction	1. Foundation damage 2. Buckling of reinforcing bars 3. Cracks in core concrete 4. Visible vertical and/or lateral deformation in columns and/or walls 5. Visible settlement and/or leaning of the buildings
5.	E	Under construction	From the laying of the foundation to the final stage
6	F	Under renovation	Any rehabilitative activity

In March 2018, MOHCDGEC launched a National Surgical, Obstetric, and Anesthesia Plans (NSOAPs). NSOAP lays out the necessary foundation to improve six major domains of the surgery, anesthesia and obstetric health care system, which are: - (a) service delivery, (b) infrastructure, (c) workforce, (d) information management and technology, (e) finance and (f) governance. This was set to address the challenge of 20% of deaths in Tanzania result from diseases that can be treated by surgical care, therefore having to ensure all Tanzanians can access safe surgical care by 2025, this has been supported also by WHO [11, 26]. In the NSOAPs, the roles and responsibilities of PORALG (Directorate of Health, Social Welfare and Nutrition Services) are as in other health plans and policies, that is includes, service implementation as well as monitoring and evaluation of the decentralization by devolution (D-by-D) policy according to the Government Notice No. 494 of 17th December 2010 as well as the Presidential Decree of 2014.

Borrowing from the education sector, a force account approach was adopted to hasten renovation process the primary health facilities. The approach is deemed cheaper compared to conventional contracting approaches. The education sector has been using this approach for construction of classes and staff houses with minimum amount of resources. Force account is the procurement method where an entity (in this case a government entity) implements rehabilitative or developmental work by utilizing its internal resources rather than contracting the work to an external entity. In such instances, the government entity may be required to procure raw materials and/or engage temporary labor to carry out the work [27, 28]. The force account is within the Tanzanian’s legal framework and procurement principles and laws [29, 30]. According to the force account approach, relevant public or semi-public agencies or departments undertake construction, when the agency has its own personnel and equipment [30]. Justification of force account is guided by clause 73-(2) [7]. The force account method is considered to be a non-competitive bid contract and an authorized local government authority like a district council, town council municipality or city council has oversight over the construction or renovation project by directly furnishing the labor, equipment, and materials [27, 30]. Japhari in 2017 [29] advanced the use of force account approach through a paper titled ‘Procedure on Effective Application of Force Account as a Method of Procurement for Renovation and Remodeling of Government Building Projects’. The paper discusses the application of force account procedures for construction and renovation of government buildings.

In this paper, we described the implementation of the innovative infrastructure improvement program for public primary health facilities from 2005 through 2019. Our study collects and analyses cross-sectional data related to status of the public health facilities at primary health care level. Specifically, we aimed at understanding the public sector’s efforts to improve the infrastructure of primary health facilities between 2005 and 2019, at the same time assessing the construction rates, geographical coverage, and physical status of the facility, safe surgery situation and services rendered in public primary health facilities.

## Methods

### Study setting

Located in East Africa, Tanzania has an estimated area of 945,087 km^2^ and a population projection (2019) of 55,890,747 (Male 27, 356,189 and Female 28,534,558). While 29.6% of the population lives in urban areas, 70.4% resides in rural localities. About 50.1% of the population is below 18 years of age, 16.2% of the population aged 5 or under, and 5.6% is aged 60 years and above [31]. As of 2019, there are 12,545 villages, 4420 Wards, 26 regions and 185 district councils in the country [31].

The health system in Tanzania operates in a decentralized system. The health care referral system is organized in a pyramid structure. At the base of the pyramid is the community, followed by dispensary, health centers and District Hospitals that constitute the primary health care. These are followed by district hospitals or designated district hospitals that are then followed by regional referral hospitals, zonal hospitals, specialized hospitals and finally the National Hospital. By December 2015, there were a total of 6640 (53%) dispensaries out of 12,545 villages (The Tanzanian policy is to have a Dispensary for every Village), of which 4554 (36%) are government owned. There are a total of 695 (15.7%) health centers out of 4420 Wards (Tanzanian policy is to have a Health Center for every Ward), of which 513 (11.6%) are government owned (Table 2). The formal distinction between dispensaries and health centers is that while dispensaries exclusively provide outpatient care, a health center should be able to provide around-the-clock care to patient and also surgical services including emergence obstetric care. The Skilled Human Resources for health gap in the health care system stands at 52% [21].

**Table 2:** Distribution of health facilities by type and ownership by December 2015

Level	Total	Type of health facility	Requirement	Health Facilities		
				Government owned	Privately owned	Total
**Village**	12,545	**Dispensaries**	12,545	4554 (36%)	2086	6640 (53%)
**Ward**	4420	**Health center**	4420	518 (12%)	183	701 (15.8%)
**District Council**	185	**District Hospital**	184	70 (65%)	38	108 (94.6%)

Infrastructure development at primary health care is coordinated by the Local Governments Authorities (LGAs) with collaboration with the local community. The central ministries (Ministry of Health and President’s Office – Regional Administration and Local Government) are responsible with provision of guidance on the building standards, structure, and equipment and provide funds for renovations of existing facilities and construction of new facilities. Currently there are about 1845 unfinished buildings of primary health care facilities in the country.

### Study design

This study relied on a cross-sectional analysis of data collected from public primary health facilities from all over the country from 2005 to 2019 as part of routine national health information on health care infrastructure and associated strategies and plans. In order to systematically appraise the processes behind the contemporary health facility improvement program of which the information analyzed in this study are based, the study was build on the following activities 1) Understanding the theory of change of the program 2) understanding the methods adopted in improving the health facilities (constructions and renovations), 3) Understanding the related reforms at various levels to support the program 4) Description of the data collection procedures and tools 5) conducting analysis of the gathered data.

### Activity 1: theory of change for infrastructure development for safe surgeries in Tanzania

Developing the theory of change (TOC) or a program theory is the first prerequisite in understanding the implementation processes and effects for any program [32]. The TOC helps to establish potential causal pathways between the primary health care development program (PHCDP) inputs and the expected outcomes that in this case is reduction in morbidity and mortality through administration of the safe surgeries.

**Fig. 1:**
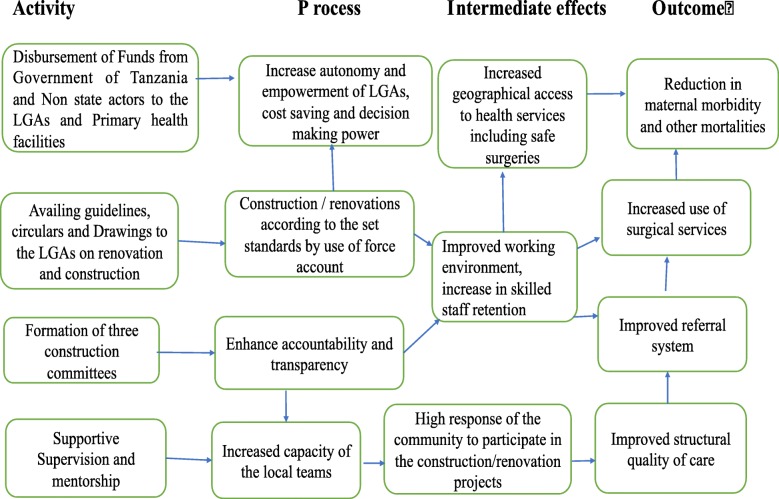
Theory of Change for infrastructure development of public primary health facilities in Tanzania

A theory of change for the extension of the Primary Health Care Development Programme (PHCDP) was conceptualized during two stakeholder meetings that included the health basket fund: during the RMOs and DMOs meeting in October 2016 and during the conclusion of the joint visit between PORALG and MoHCDGEC in December 2016 [25]. During these meetings, participants articulated the processes of change they anticipated. The authors of this paper further refined the ToC to be utilized in the prospective evaluations, based on a review of the literature (Fig. 1). Therefore, this documentation study was done so that to help understand the progress and achievement made in terms of primary health care facilities in Tanzania as an important step in the provision of safe surgeries hence reduction in surgeries related morbidities and mortalities as well as other disease conditions.

### Activity 2: methods of construction and renovations

The construction of primary health facilities that was done between January 2017 and November 2019 used force account for renovation and construction with great success. District Councils were able to procure building materials and/or engage temporary labor to carry out the work. To reduce costs and enhance community ownership, local artisans were sub-contracted by the district to renovate the buildings. District engineers were responsible for carrying out supervisions to the renovation and construction sites.

Upon receipt of funds from the central government through the Ministry of Finance and Planning, three local construction or renovation committees were created by the district were: procurement, construction as well as receipt and inspection committees. The district used the following criteria for selecting facilities for renovation: 1) A distance from the district head office of more than 20 km, 2) Health facilities with physical state designations of B, C, E or F (Table 3), meaning major or minor renovation was needed or the facility was under construction or renovation, 3) An area with a catchment population of more than 10,000 people and 4) A health center with no operating theatre. Building renovations were performed on: roofing; windows and doors; floors and ceilings; as well as plumbing, electrical, sewage, solid waste management, and water harvesting systems. In each of the district council in Tanzania, in addition to renovation, six buildings were constructed on site, including a maternity ward, outpatient department (OPD), laboratory, operating theatre, mortuary and laundry by using a force account which it has shown to be a game changer.

**Table 3:** Infrastructure elements

SN	Basic Infrastructure	Definitions
1.	Physical infrastructure	Functioning building including latrines
2.	Equipment • Building or infrastructure repair • Equipment processing	System for maintenance and repair of buildings or infrastructure
3.	Infection control • Availability of infection control items • Adequate disposal system for infectious waste	Availability of soap, running water, sharp box, latex gloves and disinfectant Collection of disposal of infectious waste No unprotected waste observed
4.	Referral system means	Presence of ambulances Presence of a reliable means of communication
5	Distance from the district council headquarters/Distance to the nearest health facilities	Less than 10 km Between 5 km -10kms More than 10 kms
6.	Safe Surgery performance	Presence of functioning operating theatre
7.	Catchment population	Number of people who are served by the health facilities

### Activity 3: understanding the related reforms at various levels to support the program

#### Organization of the efforts at the local government level

The LGAs are responsible for management and implementation of the renovation and infrastructural development program. Their responsibilities also include provision of technical support to lower levels, mainly wards and villages. The Ward Development Committees (WDC) and Village Governments (VG) are responsible for coordinating and supervising the various activities carried out at these levels. They are also responsible for mobilization of the community for their active participation and for daily supervision of ongoing projects and punishment of the local artisans. In addition, the Council Health Service Boards and Health Facility Governing Committees work together with specified committees (construction, procurement and inspection committees) to enhance accountability and governance.

#### Financing the construction/ renovation project

This program was financed from different sources within the Government of Tanzania and its implementing partners through the health basket fund. Heath Basket fund has been supporting health sector in Tanzania since the year 1999/2000, it is part of the government effort to implement a Sector Wide approach (SWAp) arrangement whereby different development partners puts their contributions into one basket and then support the health sector through 13 priority areas as spelled out in the Comprehensive Council Health Plan (CCHP) and Comprehensive Health Plans guidelines [33]. The health basket fund is considered to be one of the reliable sources of funds in the country. The release of these funds is guided by signing of the side agreements after mutual agreement between Health Basket Financing partner and Government of Tanzania and it is part of implementation of the five-year’s Memorandum of Understanding (MoU) [34]. For each healthcare facility, a total of TZS 700,000,000 (305,650.16 USD) was put aside to renovate health facilities of which TZS 500,000,000 (218,321.54 USD) was used for construction of 5 to 6 buildings (operating theatre, maternity block, laboratory, mortuary, staff building, incinerator and placenta pit) as well as waste management infrastructure and 200,000,000 (87,328.62 USD) for medical equipment (Exchange rate 1 USD = 2290.20 TZS as of 07.11.2018).

#### The direct health facility financing (DHFF) program on the renovation and maintenance of infrastructure

In 2017 the Government of Tanzania embarked on the Direct Health Facility Financing (DHFF) program with hypothesis that the program would increase provider autonomy over access to and use of resources, increase the engagement of health facility governing committees (HFGC) in the planning and financing of care, and result in the improved structural quality of care as facility resources were directly invested in service delivery [32]. Therefore, DHFF program was designed to help procure resources for renovation and maintenance of physical infrastructures at the local level and also increase accountability and value for money for all projects [32]. Since inception of construction and renovation of health facilities the DHFF program has been used as an approach for quick and reliable disbursement of funds for construction/renovation.

#### Sustainability plan of renovation and infrastructure development

In the financial year 2017/2018, the Government of Tanzania introduced a new financial mechanism that differed slightly from the traditional financial mechanism in which finances were channeled through district offices and then went to the primary healthcare facilities. In the new approach, DHFF funds go directly from the treasury to the primary health facility, which has helped to greatly reduce bureaucracy [32]. The system has also enhanced primary health facility autonomy, including over the planning and budgeting for health facilities, allowing them to make decisions regarding infrastructure development and renovations [33].

### Activity 4. Data collection procedures and tools

Data related to infrastructure development in the primary health facilities were collected from the following sources

1. Health Facility Registry (HFR): The information was obtained from the http://www.moh.go.tz/en/health_facility_registry. This provided information on the number of primary health facilities in the country and location.
2. Plans and implementation reports at different levels of the health care system. The plans include: the MMAM (2007–2017) [20], Health Sector Strategic Plan III and IV; Regional Health Management Teams (RHMT) and Council Health Management Teams (CHMT), Tanzania Service Provision Assessment (TSPA) report 2015/16 and Service Availability and Readiness Assessment (SARA) 2012 [24, 35]. The extracted information was related to physical status of health facilities in relation infrastructure and equipment.
3. Infrastructure assessment checklist: A seven-item checklist was used to examine the following elements (Table 3): physical infrastructure (latrines as well as adequate functional rooms and buildings) (Table 4) infection prevention (waste disposal facilities), and inventories for ambulances.
4. CEMONC signal functions checklist. Which assessed CEMONC nine specific signal functions for CEMONC designated facilities, such as (i) administering parenteral antibiotics, (ii) administering uterogenic drugs for active management of the third stage of labour and prevention of postpartum haemorrhage, (iii) use of parenteral anticonvulsants for the management of pre-eclampsia/eclampsia, (iv) manual removal of placenta, (v) removal of retained products of conception (e.g. manual vacuum extraction, dilatation, and curettage), (vi) performing assisted vaginal delivery (AVD), i.e. vacuum extraction or forceps delivery, (vii) performing basic neonatal resuscitation), (viii) performing CS delivery, and (ix) Safe Blood Transfusion services to be available for 24 h a day, 7 days a week [36–38].

**Table 4:** Construction and upgrading of dispensary and health center buildings by using force accounts, up to August 2019

Year	Total number of dispensaries constructed	Average Annual Rate of Increase (Dispensaries)	Total number of Health Centers constructed	Average Annual Rate of Increase (Health Centers)	Number of dispensaries renovated and upgraded	Number of health centers renovated/ constructed	Number of staff buildings constructed
2005	3038	0.031	333	0.046	0	0	0
2010	3550	0.050	420	0.042	0	0	0
2015	4554	0.016	518	0.048	0	0	0
2019	4922		716		181	350^a^	390

^a^Construction of health centers, outpatient departments, operating theatres, laboratories, pediatric and maternity wards, as well as waste management facilities like incinerators and placenta pits

### Activity 5: conducting analysis of the gathered data

#### Variable and measures

In this study, the variables of interest were:

Health facility status variables which included: 1) Type of a health facility, categorized into dispensary and health center 2) Distance of the health facility from district headquarters was measured in kilometers 3) years of operation was measured years and categorized in 5 years intervals 4) Training status on maternal health and safe surgeries 5) Physical status of the facility categorized into A to F 6) location of the health facility measured as urban or rural.

CEMONC services data: measured in relation to presence or absence of CEMONC services

Amenities in health facilities; the amenities were divided into physical utilities and waste management equipment. In this category variables were measured into yes or not in relation to availability of a given amenity. The amenities studies include: Water 2) Electricity 3) Phone 4) incinerator 5) Placenta and 6) Waste bin.

### Statistical analyses

We used MS Excel spread sheet (Microsoft Excel®, Microsoft Corporation) to manage the collected data and thereafter imported to Statistical Package for social science (SPPS) program version 22 (SPSS Inc., Chicago, IL, USA) for further analysis. Descriptive statistics were used to summarize data, some of them where frequencies, percentages and then followed by inferential statistics by using a chi-square and bivariate analysis to determine relationship between variables and location of the health facility (rural vs urban).

## Results

### Demographic characteristics

A total of primary health facilities 5072 (4554 Dispensaries and 518 Dispensaries) were constructed cumulatively by December 2016 (Table 2). Of these health facilities 4566 (90%) were constructed in rural settings and 506 (10%) were constructed in the urban settings. A total of 2159 (42.5%) of people were residing between 5 and 10 kms from the health care facility (Table 5). There has been a average annual increase in the number of Health centers constructed as compared to the dispensaries were there have been deceleration since 2015. Between 2015 and August 2019 a total 508 primary health were constructed and renovated, including 350 Health centers and 69 District Council Hospitals. This was accompanied with constructions of 390 staff houses.

**Table 5:** Descriptive results of public primary health facilities by settings (urban and rural) (*N* = 5072) up to December 2015

Variable	Description	Rural, n (%)	Urban, n(%)	Total (%)	Chi-square	***P***-value
**HF level**	Dispensary	4091(90)	463(87.4)	4554 (90)	5.39	0.015
	Health Centre	451(10)	67(12.6)	518 (10)		
**Distance from the nearest facilities (kms)**	< 5	1095(24)	365(72)	1369 (27)	242.63	< 0.0001
	5–10	2190(48)	112(22)	2159 (42.5)		
	11–20	867(19)	15(3)	857 (16.9)		
	> 20	411(9)	15(3)	685 (13.5)		
**Years of operation**	< 5	548(12)	39(7.7)	507 (10)	2.39	0.124
	5–10	821(18)	96(18.9)	811 (16)		
	> 10	3194(70)	373(73.4)	3752 (74)		
**Training Status**	< 50%	1679(51.8)	103(28.1)	2282 (45)	72.23	< 0.0001
	50%+	1563(48.2)	264(71.9)	2790 (55)		
**Functionality of Health Centers**	HCs with CEMONC services	70 (15.5)	45(67.2)	115 (22.2)		
	HCS without CEMONC services	381(84.5)	22(32.8)	403 (77.8)		
**Physical Status**						
**Water available**	Yes	1456(31.9)	175(34.5)	1673	0.983	0.161
	No	3107(68.1)	332(65.5)	3397		
**Electrical power available**	Yes	1939(42.5)	197(38.8)	2129	1.943	0.09
	No	2624(57.5)	310(61.2)	2941		
**Phone availability**	Yes	196(4.3)	62(12.2)	1775 (55)	45.41	< 0.0001
	No	4367(95.7)	445(87.8)	3295 (65)		
**Medical waste management**						
**Incinerator available**	Yes	903(19.8)	134(26.4)	1034 (20.4)	9.53	0.002
	No	3660(80.2)	373(73.6)	4036 (79.6)		
**Placenta Pit available**	Yes	1561(34.2)	132(33.4)	1693 (33.4)	10.41	0.001
	No	3002(65.8)	375(73.9)	3377 (66.6)		
**Waste bin availability**	Yes	101(2.2)	18(4.6)	127(2.5)	8.14	0.007
	No	4462 (97.8)	376(95.4)	4943 (97.5)		
**Physical Status**	A-B	3578(78.4)	412(81.2)	4005 (78.9)	0.514	0.278
	C-G	986(21.6)	95(18.8)	1065 (21.1)		

A = good

B = minor renovation needed

C = major renovation needed

D = demolition and reconstruction

E = under construction

F = under renovation

### Health facility physical status

Majority (46%) of primary health care facilities had a physical status of A (good state) while 33% (1693) had physical status of B (minor renovation needed) and the remaining facilities had physical status of C up to F (Table 5). Majority (70%) of primary health facilities have been operating for more than 10 years.

### Comprehensive emergence obstetric care

Of all (518) Health centers, only 115 (22.2%) were offering the Comprehensive Emergence Obstetric Care (CEMONC). It was found that out 115 health facilities only 20 (17.4) were offering the CEMONC with all 9 - signal functions [28, 29].

### Amenities

In this study only 17.4% of 115 health centers had facilities for offering blood transfusion services and 33% (1673) of health facilities had piped water and 5.1% had landline telecommunication system (Table 2).

**Fig. 2:**
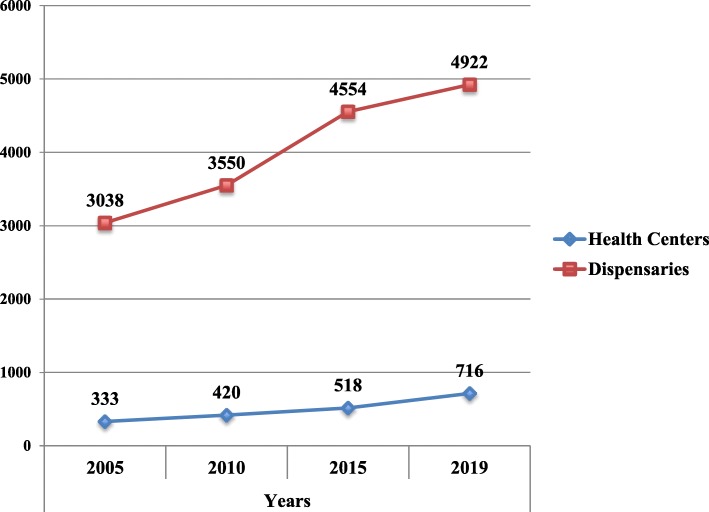
Health center and dispensary construction trends from 2005 to 2019

**Fig. 3:**
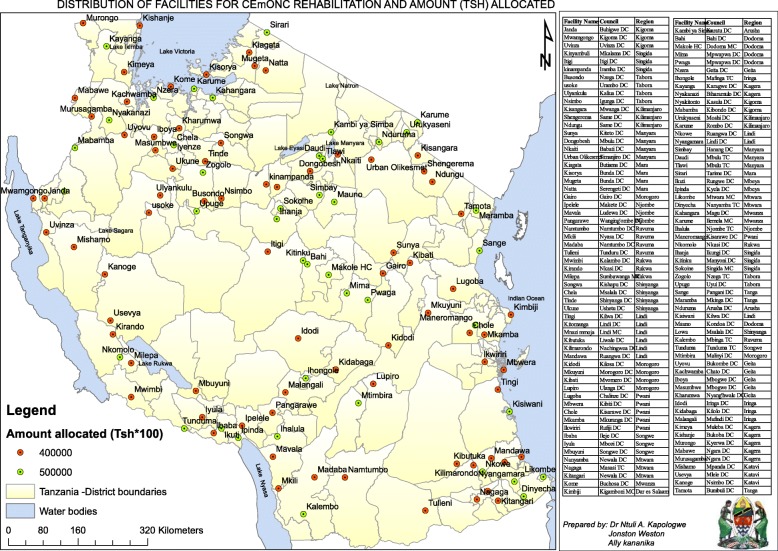
Map of Tanzania that depicts the distribution of health facilities offering CEMONC services (Prepared by Kapologwe NA, Wenston J & Kananika A). Sources: Shape files were obtained from Tanzania National Bureau of Statistics (NBS) that was updated in 2016. The used software was Arc Geographical Information System (Arc GIS) version 10.3. Device used to collect spatial data was Global Positioning System (GPS)

The trend of construction and renovation has shown a sharp increase in the construction of buildings between 2017 and 2018 (Fig. 2). The construction and renovation projects were implemented across the country (Fig. 3) basing on the set criteria (Tables 1 and 3). In addition to the above criteria, it was agreed that local artisans were used, while adhering to the procurement act and principles of force account approach.

Review of reports submitted from Regions and District Council indicated that out that; there were 1845 unfinished primary health facilities buildings across the country and they were at different levels of construction.

Bivariate analysis using chi-square test, we found that facility location was significantly associated (*P* < 0001) with receipt of more training on basic surgical services as those who were working in the rural public primary health facilities received more training than the rural ones (Table 5). We also found that distance to the Public primary health facilities to be significantly associated with location of facilities (*P* < 0.0001) as people who are resides in the urban have access to their facilities within 5 KMs as oppose to those who are in rural (Table 5).

## Discussion

This study assessed public primary health facility infrastructure development in Tanzania in the context of improved access and equity to surgical health care as a strategy towards achieving Universal Health Coverage (UHC). The majority (46%) of primary health care facilities had a physical status of A (good state) while 33% (1693) had physical status of B (minor renovation needed) and the remaining facilities had physical status of C up to F. This means that majority of facilities were offering sub optimal quality of health care services, as their amenities were not up to the standard.

The finding that only 33% (1673) of health facilities had running water indicates that hygiene and sanitation is still a challenge in most of health care facilities hence making the provision of quality of health services in the health care facilities a question. This finding is similar to the study that was done by WHO which showed 42% of all assesses health care facilities in African region were lacking improved water sources [39].

In this study we found that only 5.1% had a telecommunication system. This impairs the ability to implement referral systems between health care facilities and providers. A study done in Ethiopia showed that, 38.1% of studied primary health care facilities had no fixed or mobile telephone services [39, 40]. To improve communication in these settings there is a need for public private partnership to allow the private sector to help in the installation of telecommunication systems in the health facilities. Rapid, affordable communication is corner stones for proper patient communication, referral, treatment and follow up [41].

From this study, we learned that approximately 54% of health facilities in Tanzania needed some sort of renovation or reconstruction before December 2016 in order to offer the quality health services to its catchment populations. Some of these facilities moved from needing minor renovations to needing major renovations due to lack of renovation or rehabilitation plan and also lack of planned maintenance culture in many existing health facilities. Scholz et al. [37] when assessing primary health facilities in Tanzania reported similar results. The importance of sustainable maintenance of health facilities may help patients to trust the normal referral system as all necessary services at their localities. In 2010, 70% of patients attended at the Muhimbili National Hospital, 67% of presented with surgical conditions and 96% stated the reason for self-referral was lack of expertise at the district hospital and primary health care facilities [42]. This highlights the importance of planned preventive maintenance at the primary health care level. However, the introduction of the Direct Health facility Financing (DHFF) in 2017 offers an opportunity for primary health facilities to plan for routine preventive maintenance into their annual budget and plans and sustain these plans in subsequent years [32].

Findings from this study, revealed that between 2015 and 2019, there were renovations or construction of 350 health centers and 69 district Hospitals with ability to offer safe surgical services through the use of a force account approach, whereby in this aspect, the Local Government Authorities implemented rehabilitative or developmental work by utilizing its domestic resources rather than following the conventional contracting processes. This approach made possible to construction of 390 staff houses which were build along with health facilities [43]. Use of force account as an approach for construction or renovation of primary health care facilities has resulted into saving of money, as there was construction of more than 5 health facility buildings instead of two buildings that would have been constructed under a traditional contracting approach. Japhari [29] also reported the benefits of using force account in the similar situation. The majority (90%) of facilities constructed before 2016 are in the rural settings, making the urban population being over reliant to the private sector.

This finding is similar to finding obtained in the study done by Nyberger et al. [44] on the situation of safe surgery and anesthesia in Tanzania. There is a need to have adequate investment in the urban infrastructure at the same time having strong Public-Private Partnership framework that will help the people who are residing in urban areas to have subsidized cost of health care.

From the desk review data, there are still 1845 unfinished primary healthcare buildings across the country. Completion of these facility buildings is critical so that they can start offering services while helping to improve the geographical access to the people. This should be done through the Sector Wide Approach (SWAp) that was used for construction of 350 Health centers.

This study also identified that; only 115 (22.2%) of health centers were providing CEMONC services and the remaining health centers were not offering the said services due to several reasons including lack of space, medical equipment and skilled personnel. And of those 115 health facilities, only 20 (17.4) were offering all 9 - CEMONC signal functions [36, 42] and only 17.4% were offering safe blood transfusion services. To provide safe obstetric care, it is very important that all 9-signal functions should be present in all CEMONC facilities. Therefore, there is a need to invest more on training and more investment on the safe blood transfusion services. The findings from this study is similar to that of Roy et al. [38] which was conducted in Bangladesh which showed that of all signal functions coverage for both Caesarean section and blood transfusion services had less than 1%.

The facility location was significantly associated with receipt of more training on basic surgical services as those who were working in the rural public primary health facilities received more training than the rural ones. This might be due to fact that those who reside in urban areas are considered to have some other means to getting information. Distance to the Public primary health facilities is significantly associated with location of facilities as people who are residing in urban areas were found to have access to their facilities within 5 KMs as oppose to those who are in rural. This might be due to fact that most villages have scattered population as oppose to those in the urban.

This will be needed for the Tanzanian setting as well to have a better outcome for surgeries conducted to the well-constructed health facilities. Surgery has become a global health priority with the adoption of the World Health Assembly Resolution 68.15 that called for the strengthening of emergency as well as essential surgical and anesthesia care as an integral component of Universal Health Coverage (UHC) [27, 44].

Maternal health can be improved as a result of quality infrastructure development in many countries; therefore, Tanzanian road map for infrastructure improvement should be continuously monitored and evaluated so to ascertain its contribution. This is something which is also explained in the report of WHO of 2016 on network for improving quality of care for maternal, newborn and child health: Monitoring framework [45].

From the document review, still there are 3821 geographical wards without any health centres and 6005 villages without dispensaries. Then need for thorough analysis and informed prioritization of future construction is needed to address the existing gap. This should go hand in hand with mobilization of domestic funds like improved Community Health Funds (CHF) that can be used for development projects like renovation or construction as it has shown to be performing well in some parts of Tanzania [46, 47].

Despite all these efforts that are geared towards Universal Health Coverage it is still very important to match facility scale up with human resource mobilization to meet expectations by having realistic population to facility relationships. This should be coupled by deployment of skilled staff at the primary health facilities that have already been constructed as the shortage of skilled health workforce is at 52% [48, 49]. Moreover, all constructed health facilities should be equipped with medical equipment that are relevant to the constructed/renovated health facilities so that to make them functional so that to increase health system responsiveness by patient who are visiting primary health care facilities as it is still low at 69.1% [50].

Safe surgery 2020 needs good infrastructure as a part of structural quality but this needs to go hand in hand with other components like skilled health personnel such as anesthetists, medical doctors and nurses, medical equipment and supplies as well as safe blood transfusion practices.

### Methodological consideration

This study has several strengths one of which is it involved data collection from the entire country and also it is the first of this kind to be conducted in Tanzania, therefore lies the foundation for further studies and evaluation in the subject in question. However, this study is limited in the sense that; it didn’t involve any questionnaire administration or interviews to the study subjects rather it relied mainly on submitted reports from the available tools for data collection. The other limitation is that; this study mainly focused on amenities although and less on the outcome of the patients. The checklist that was used for assessment is mainly limited to few areas in the basic amenities to Health centers and Dispensaries and offers little to the district hospitals.

## Conclusions

Geographical access to the health service is important for the access to safe surgeries and attainment of Universal Health Coverage at large. Due to the fact that, resources are constraints and the fact that still the demand for new health facilities construction is high, it is therefore, very important that renovation and construction should be done so that to demand of the public while maintaining the value for money. Moreover, it is high time to incorporate all infrastructure data into existing health management information system like DHIS-2 so that they can be easily accessed, analyzed and hence evaluation of ongoing infrastructural development projects.

Potential take away points:

1. Infrastructure is necessary for safe surgery and was part of the NSOAP domains for Tanzania and this is critical to address to achieve UHC.
2. Rapid scale up is possible with a force account approach that speeds up construction.
3. HIMS systems like DHIS-2 must be utilized to track infrastructure projects.

## Data Availability

It is declared that data are available and obtainable in the established database housed at the Ministry of Health, Community Development, Gender, elderly and Children through http://hfrportal.moh.go.tz/ and President’s Office – Regional Administration and Local Government through http://hssrc.tamisemi.go.tz/. All additional raw data can be obtained from the corresponding author upon request, while other data have been presented in the tables, maps and figures of this paper.

## Abbreviations

BG: Block Grant
CHMT: Council Health Management Team
DED: District Executive Director
DHIS-2: District Health Information System − 2
DMO: District Medical Officer
HFGC: Health Facility Governing Committee
HMIS: Health management information system
Kms: Kilometers
LGCDG: Local Government Capital Development Grant
MoHCDGEC: Ministry of Health Community Development, Gender, Elderly and Children
MOHSW: Ministry of Health and Social Welfare
PO-RALG: President’s Office – Regional Administration and Local Government
PPHCF: Public Primary Health Care Facilities
RAS: Regional Administrative Secretary
RBF: Results Based Financing
RHMT: Regional Health Management Team
SS: Safe surgery
ToC: Theory of change
